# Age Effects on Spatiotemporal Dynamics of Response Inhibition: An MEG Study

**DOI:** 10.3389/fnagi.2018.00386

**Published:** 2018-11-20

**Authors:** Mei-Yin Lin, Yi-Jhan Tseng, Chia-Hsiung Cheng

**Affiliations:** ^1^Department of Occupational Therapy and Graduate Institute of Behavioral Sciences, Chang Gung University, Taoyuan, Taiwan; ^2^Laboratory of Brain Imaging and Neural Dynamics (BIND Lab), Chang Gung University, Taoyuan, Taiwan; ^3^Department of Physical Medicine and Rehabilitation, Taichung Hospital, Ministry of Health and Welfare, Taichung, Taiwan; ^4^Department of Medical Research, Hsinchu MacKay Memorial Hospital, Hsinchu, Taiwan; ^5^Healthy Aging Research Center, Chang Gung University, Taoyuan, Taiwan; ^6^Department of Psychiatry, Chang Gung Memorial Hospital, Linkou, Taiwan

**Keywords:** inhibition, aging, magnetoencephalography (MEG), Go/No-go task, executive functions

## Abstract

Inhibition, the ability to suppress irrelevant information, thoughts or movements, is crucial for humans to perform context-appropriate behaviors. It was suggested that declined cognitive performance in older adults might be attributed to inhibitory deficiencies. Although previous studies have shown an age-associated reduction in inhibitory ability, the understanding regarding its cortical spatiotemporal maps remained limited. Thus, we used a whole-head magnetoencephalography (MEG) to elucidate the age effects on response inhibition, and to explore the brain activation differences in high- and low-performing seniors. We recruited 22 younger and 22 older adults to participate in the visual Go/No-go task. Both behavioral performance and neuromagnetic responses to No-go stimuli were analyzed. The behavioral results showed that the older adults made more false alarm (FA) errors than the younger adults did. The MEG results showed that the seniors exhibited declined cortical activities in middle temporal gyrus (MTG) and delayed activation in MTG, prefrontal cortex (PFC) and pre-supplementary motor area (pre-SMA). Furthermore, among the older adults, more recruitment of the left PFC was found in the high-performers than in the lower-performers. In conclusion, age-related deficiencies in response inhibition were observed in both behavioral performance and neurophysiological measurement. Our results also suggested that frontal recruitment plays a compensatory role in successful inhibition.

## Introduction

Response inhibition is a core executive function of humans to perform functional activities in everyday lives. Successful goal-oriented tasks require the brain’s ability to suppress irrelevant information, thoughts or behaviors in order to focus on task-relevant demands (Collette et al., 2009). Many studies have indicated that failure in inhibition is one of the manifestations in neurodegenerative or psychological disorders, such as Alzheimer’s disease (Collette et al., 2009; Cheng et al., 2012), mild cognitive impairment (Cid-Fernández et al., 2014), Parkinson’s disease (Dirnberger and Jahanshahi, 2013), depression (Kaiser et al., 2003) and anxiety (Robinson et al., 2013). In addition, response inhibition has been found to be modulated by physiological aging processes (Turner and Spreng, 2012). Several lines of evidence have shown that inhibitory ability not only influences the prospective memory performance (Schnitzspahn et al., 2013), but also affects speech production and verbal fluency in older adults to maintain effective communications (Bricker-Katz et al., 2009; Shafto and Tyler, 2014). However, it should be noted that inhibitory deficiency is not an inevitable result of advancing age (Andrés et al., 2008; Collette et al., 2009; Pires et al., 2014). It has been asserted that controlled or top-down inhibitory processes are more vulnerable to brain aging, while automatic inhibitory function, which occurs before conscious awareness, is relatively preserved (Andrés et al., 2008; Collette et al., 2009).

The Go/No-go task has been well-established and extensively used to study the restraint component of inhibition (Vara et al., 2014). Several studies have examined the inhibitory processes in normal aging using behavioral and electrophysiological measures. For example, it has been shown that, behaviorally, the older adults spent longer time to respond to a target but had similar accuracy compared to the younger adults (Falkenstein et al., 2002; Hsieh et al., 2015; Mudar et al., 2015; Staub et al., 2015). In terms of electrophysiological results, some studies have demonstrated an age-related reduction in neural activations (Tachibana et al., 1996; Bokura et al., 2002; Hsieh et al., 2015; Mudar et al., 2015; Staub et al., 2015), whereas others did not find such difference between younger and older adults (Falkenstein et al., 2002; Vallesi et al., 2009; Hsieh et al., 2016). Moreover, it has been found that compared to the younger participants, the older participants showed more cortical activities in the frontal regions (Fallgatter et al., 1999; Vallesi et al., 2011; Hong et al., 2014; Staub et al., 2014). Taken together, the exact profile of age-related cortical changes in response inhibition remains controversial. Up to the present, there was limited literature to elucidate the neural correlates of age-related alterations in the inhibitory processes of action restraint. Hence, the first aim of this study was to utilize a whole-head magnetoencephalography (MEG) and Go/No-go task to explore the age-associated changes in response inhibition at the cortical level.

Previous studies have shown that not only general declination but also increasing inter-individual variability contributed to age-related alterations in cognitive performance (Li and Lindenberger, 1999; Christensen, 2001; Hultsch et al., 2002). These findings support a prominent theory to elucidate that the defective inhibitory function might be a fundamental factor to reflect the variability of cognitive performance, e.g., high-performers vs. low-performers, among older adults (Hasher and Zacks, 1988). However, the neural activation of response inhibition between the high- and low-performing seniors were less studied. With these concerns, we further divided the older participants into high- and low- performers based on the behavioral performance of response inhibition (i.e., the false alarm (FA) rate) to explore their differences in brain activation patterns, which was the second aim of this study.

Compared to electroencephalography (EEG), MEG has superiority in localization of cortical activities. Thus, the present study aimed to apply MEG combined with distributed source modeling methods to examine the age-related changes of the neural correlates in the Go/No-go paradigm. In the aged group, we further explored the differences in cortical activation between the high- and low-performers. We hypothesized that older adults would show poorer behavior performances compared to younger adults in our task, and age-related declinations in brain activation would be observed in the MEG recordings. Moreover, it was hypothesized that there would be significant differences in source activations between the high- and low-performers in the older group.

## Materials and Methods

### Subjects

Forty-four healthy volunteers participated in the study. All the subjects confirmed that they were right-handed, free of history of neurological and psychological disorders, and had normal or corrected-to-normal vision with contact lenses. Also, we excluded the participants with risks of developing dementia by using the Cognitive Abilities Screening Instrument (Lin et al., 2012).

Twenty-two were assigned to the younger (aged 20–28 years, 11 females), and 22 were assigned to the older (aged 55–73 years, 14 females) groups. The details and comparisons of demographic data between two groups were listed in Table 1. This study was approved by the Institutional Review Board of Taipei Veterans General Hospital (Taipei, Taiwan), and was performed in accordance with approved guidelines and regulations. The written informed consent was obtained from all the participants after detailed descriptions of experimental procedures.

**Table 1:** Characteristics of the participants.

	Young	Elderly	*p*-value
*N*	22	22	-
Gender (female:male)	11:11	14:8	0.361
Mean age (SEM)	24.41 (0.40)	62.41 (0.97)	<0.001*
CASI score (SEM)	96.77 (0.62)	93.55 (0.80)	0.003*

SEM, standard errors of the mean; CASI, Cognitive Abilities Screening Instrument. *p < 0.05.

### Experimental Design and Procedure

All the participants were comfortably seated in a magnetically shielded room, with his/her head supported by the helmet-shaped device of MEG (Vectorview, Elekta-Neuromag, Helsinki, Finland). Their right index fingers were placed on a small plate with a light-emitting diode sensor to record the behavior responses. The subjects were instructed to respond to Go stimuli as accurately and quickly as possible, and withhold responses to No-go stimuli. We provided the subjects with a sufficient set of practice (at least 20 trials) to ensure that they were familiar with the content of the task.

**Figure 1:**
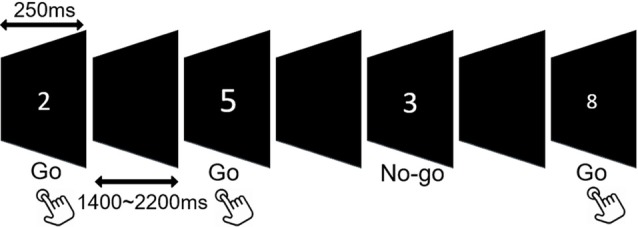
Schematic illustration of the Go/No-go task.

In the Go/No-go paradigm, digits ranging from “1” to “9” were presented at the center on a projector screen in a quasi-random order at a distance of 60 cm. The stimuli were varied in font sizes of 67–120 pt to prevent perceptual factors of target numbers from inducing bottom-up attentional modulation (Dippel et al., 2016). The stimulus duration was 250 ms, and the inter-stimulus interval (ISI) varied randomly between 1,400 ms and 2,200 ms to prevent the anticipation effects. Participants were instructed to respond to each digit (Go) except for numbers “3” and “7” (No-go), which required response inhibition. The experiment comprised at least 450 trials including 315 Go (70%) and 135 (30%) No-go stimuli (Figure 1).

The reaction time (RT), miss rate and FA rate were recorded for further analysis. We measured the RT from the stimulus onset to the participants’ response. Only the RTs to Go trials between 200 ms and 1,000 ms were included. Miss rate is defined as the ratio of missing Go responses to the total number of Go trials. A lower miss rate is indicative of a higher attention level during the whole experiment. FA rate is a measure to infer the magnitude of disinhibition. It is estimated as the number of incorrect No-go responses divided by the total number of No-go trials. A higher FA rate indicates a reduced inhibitory performance.

### MEG Acquisition and Pre-processing

A 306-channel whole-head MEG device was used to record neuromagnetic responses. We analyzed and plotted the event-related fields from the 204 planar gradiometers which detect the maximal signals from the sources and reduce external magnetic disturbances (Hämäläinen et al., 1993). Prior to MEG recordings, four indicator coils and three anatomical fiducial points (nasion and bilateral preauricular points) were marked to determine the precise location of the head by the three-dimensional digitizer. Additional head points uniformly distributing on the head surface were also digitized. The indicator coils were connected with the MEG to decide the head position. The fiducial points and other head points provide the spatial information to integrate the MEG data into default anatomy.

At least 70 artifact-free correct No-go trials were acquired and averaged in each group. The MEG recording epoch was 900 ms including 100 ms pre-stimulus baseline. We removed the artifacts of eye blinks (EOGs >300 μV) using signal space projections (SSPs) and excluded the epochs which contained amplitudes over 2 pT from any sensor. The online bandpass filter was set at 0.1–200 Hz with a sampling rate of 600 Hz. An offline bandpass filter was set at 1–40 Hz to prevent contaminations of muscle artifacts (Blanco-Elorrieta and Pylkkänen, 2016).

### Data Analysis and Statistics

#### Behavioral Data

We analyzed RT, miss rate and FA rate by using independent *t*-tests to measure the differences between the younger and older groups. Besides, the older adults were split into two subgroups (50%, 50%) according to the FA rate, and then we compared the behavioral differences between high- and low-performers by using independent *t*-tests.

#### MEG Data

An overlapping sphere model was applied to calculate the external magnetic fields to match the head shapes according to the strength of the electric dipoles (Huang et al., 1999). Next, we applied depth-weighted minimum norm estimate (wMNE) to analyze the cortical spatiotemporal dynamics of the recorded magnetic responses with the Brainstorm software (Tadel et al., 2011). The approach was mathematically expressed as the L2-minimum norm estimates of currents density in the brain to localize distributed cortical activations (Hämäläinen and Ilmoniemi, 1994; Larson and Lee, 2014). Furthermore, cortically constrained MNE was estimated with the source space comprising of 15,000 elementary current dipoles to show individual networks on ICBM 152 brain template by Brainstorm’s registration methods.

**Figure 2:**
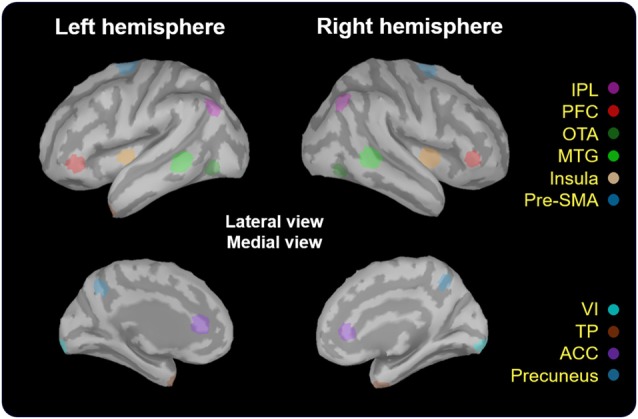
Localization of regions of interest (ROIs). These regions (5–8 cm^2^) were scouted on ICBM 152 brain template. TP, temporal pole; IPL, inferior parietal lobule; PFC, prefrontal cortex; OTA, occipitotemporal area; MTG, middle temporal gyrus; Pre-SMA, pre-supplementary motor area; VI, primary visual cortex; ACC, anterior cingulate cortex.

The estimates of baseline noise of each dipole were transformed into z-score maps, providing the statistical reliability of the signal at each location (Cheng et al., 2015). Based on the grand-averaged waveforms of the obtained data, we mainly extracted the time window from 200 ms to 600 ms after the onset of No-go trials, corresponding to the temporal processing of response inhibition (Vidal et al., 2012; Vara et al., 2014). The wMNE activation of each participant was averaged onto the ICBM 152 cortical surface. The selection of regions of interest (ROIs) was based on the grand-averaged wMNE source maps from 200 ms to 600 ms in our study. Each cortical vertex was manually scouted referring to the template of Desikan-Killiany and Brodmann area in the Brainstorm software to define ROIs. Each scout was set to cover 40 vertices, corresponding to 5–8 cm^2^. The maximal activation cluster in each ROI was used as a center of the scout. In order to reflect the potential neural activation of the No-go network, we also referred to previous studies to determine the possible brain regions as a functional ROI (Criaud and Boulinguez, 2013). For instance, in the prefrontal area, the inferior frontal cortex was the region which was pertinent to response inhibition (Aron et al., 2004, 2014). Therefore, we chose the prefrontal cortex (PFC; approximately the inferior frontal region) as a functional ROI for cortical activities of inhibition. Finally, we determined to examine the following ROIs: bilateral temporal pole (TP), inferior parietal lobule (IPL), PFC, occipitotemporal area (OTA), middle temporla gyrus (MTG), insula, pre-supplementary motor area (pre-SMA), primary visual cortex (VI), anterior cingulate cortex (ACC) and precuneus (Figure 2). The peak amplitude and peak latency were obtained within the pre-defined time window in each ROI in every participant for the further analyses.

#### Statistical Analyses

All the data were presented as mean ± standard error of the mean (SEM). We conducted two-factor mixed-design ANOVAs [within-factors: HEMISPHERE (left and right hemispheres) and AGE (younger and older adults)] to explore age-related differences in brain responses (i.e., peak amplitude and peak latency) in the No-go trials. In additionn, we compared the high-performers to low-performers within the older group by two-way repeated measured ANOVAs (HEMISPHERE × GROUP) to resolve the inter-individual differences of brain responses.

## Results

### Comparisons Between Young and Old Adults

There was no significant between-group difference in RT (*t*_(42)_ = 0.072, *p* = 0.943) and miss rate (*t*_(42)_ = −0.938, *p* = 0.354), while the older adults (M ± SEM = 17.91 ± 2.66%) had significantly more FA errors (*t*_(42)_ = −2.387, *p* = 0.022) than the younger adults did (10.17 ± 1.85%; Table 2).

**Table 2:** Behavioral performance between younger and older groups.

	Young		Elderly	
Variables	Mean	(SEM)	Mean	(SEM)	*t*	*p*-value
RT (ms)	446.24	(11.37)	444.89	(14.91)	0.072	0.943
Miss rate (%)	1.14	(0.41)	1.73	(0.48)	−0.938	0.354
False alarm rate (%)	10.17	(1.85)	17.91	(2.66)	−2.387	0.022*

RT, reaction time; SEM, standard errors of the mean; *p < 0.05.

**Figure 3:**
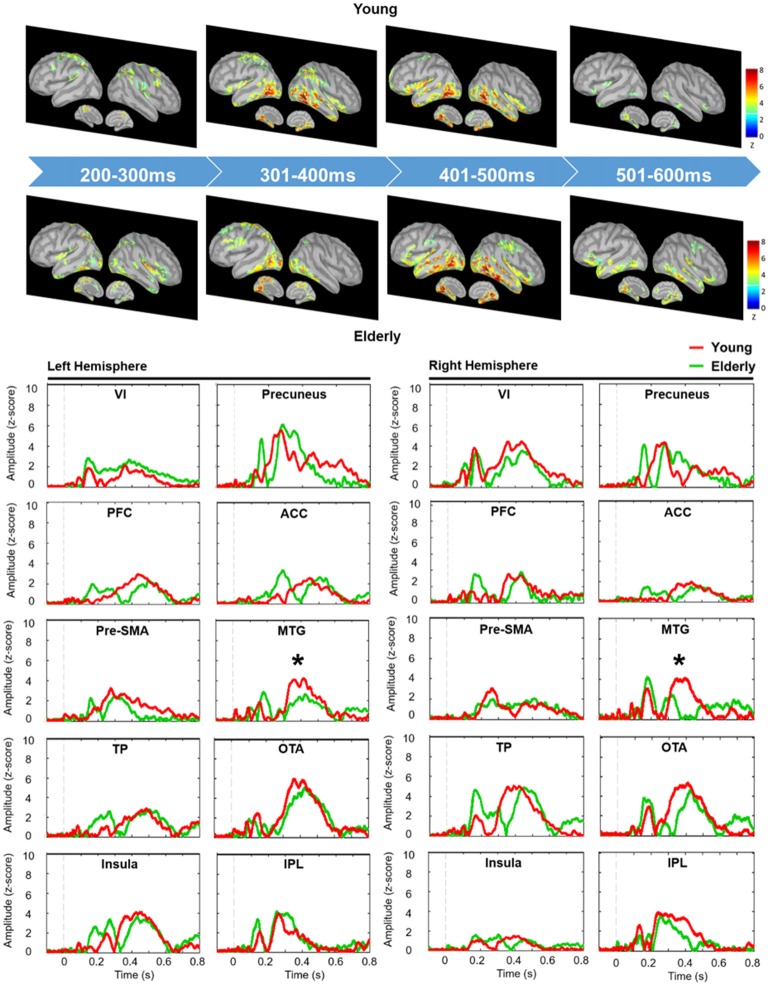
Grand-averaged spatiotemporal dynamics (upper panel) and depth-weighted minimum norm estimate (wMNE) source waveforms (lower panel) of No-go trials in each group. Each wMNE cortical map was obtained by averaging cortical responses across every 100-ms time window from 200 ms to 600 ms. Source waveforms of each ROI were displayed from −100 ms to 800 ms in the younger (red traces) and older (green traces) groups. Asterisk represents the significant differences of peak amplitude. VI, primary visual cortex; PFC, prefrontal cortex; Pre-SMA, pre-supplementary motor area; TP, temporal pole; ACC, anterior cingulate cortex; MTG, middle temporal gyrus; OTA, occipitotemporal area; IPL, inferior parietal lobule.

**Figure 4:**
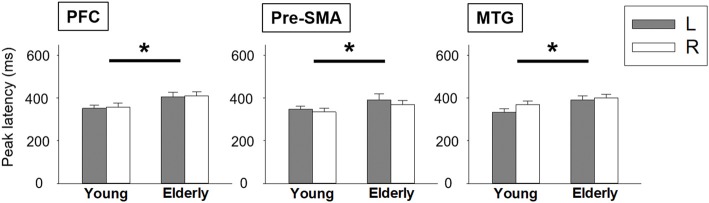
Statistical comparisons of peak latency between younger and older groups. Error bars represent the standard errors of the mean (SEM). *p < 0.05. L, left hemisphere; R, right hemisphere; PFC, prefrontal cortex; pre-SMA, pre-supplementary motor area; MTG, middle temporal gyrus.

We depicted the source imaging maps between 200 ms and 600 ms after the onset of Nogo stimuli. The grand-averaged spatiotemporal dynamics and source waveforms in the younger and older groups were presented in Figure 3. On the whole, older adults showed reduced cortical activations and more widespread brain regions, such as frontal area, compared to the younger group. Further analysis showed that the peak amplitude of MTG was significantly reduced in the older participants (*F*_(1,42)_ = 8.470, *p* = 0.006). Moreover, we found that compared to the younger participants, the older adults showed significantly delayed peak latency of PFC (*F*_(1,42)_ = 8.080, *p* = 0.007), pre-SMA (*F*_(1,42)_ = 5.305, *p* = 0.026) and MTG (*F*_(1,42)_ = 5.914, *p* = 0.019; Figure 4). No significant difference was found with the factor of HEMISPHERE.

### Comparisons Between High- and Low-Performers of the Older Adults

**Figure 5:**
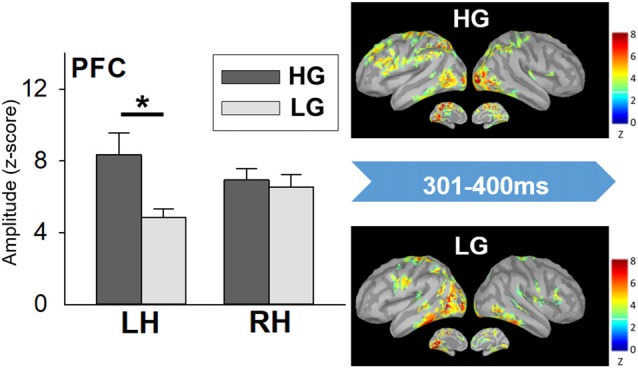
Cortical spatiotemporal map during 301–400 ms (left panel) and PFC amplitude (right panel) in responses to No-go stimuli between high- and low-performers. Error bar represented the SEM. *p < 0.05. PFC, prefrontal cortex; LH, left hemisphere; RH, right hemisphere; HG, high-performing group; LG, low-performing group.

The statistical results did not show significant differences in miss rate between the high- and low-performers. High-performers (485.74 ± 21.46 ms) spent longer RT to Go trials than low performers did (404.04 ± 11.82 ms; *t*_(20)_ = 3.334, *p* = 0.003). A significant interaction effect was apparent in the PFC (*F*_(1, 20)_ = 7.555, *p* = 0.012). The further analysis showed that high-performers had stronger neural activation in the left PFC than low-performers did in the time window of 301–400 ms (*t*_(20)_ = 2.677, *p* = 0.014; Figure 5). No other significant difference was found.

## Discussion

In the study, we examined the age-associated alterations of brain activation to response inhibition during 200–600 ms by MEG recordings, and then further analyzed the cortical activation differences between high- and low-performers in the older participants. The results demonstrated defective inhibition in the aging brains. Furthermore, we found that the high-performing seniors recruited more frontal sources than low-performing seniors to compensate for their behavioral manifestations.

Compared to the younger adults, the older adults demonstrated a significantly higher FA rate in the present study, suggesting that the seniors indeed demonstrated a relatively declined inhibitory function to withhold their responses. Our behavioral data were in line with previous study, showing that seniors made more FA errors than youngers did (Hsieh et al., 2016); however, several studies indicated that older adults had comparable FA rates to younger adults’ (Falkenstein et al., 2002; Vallesi et al., 2009; Vallesi, 2011; Hong et al., 2014; Staub et al., 2014, 2015; Hsieh et al., 2015; Mudar et al., 2015). The possible reason to account for the discrepancies may be the inconsistent experimental settings across studies. Some of these studies used easier tasks by equal probability of Go and No-go stimuli (Falkenstein et al., 2002; Vallesi et al., 2009; Vallesi, 2011; Hong et al., 2014), yielding the results with no age-related differences in FA errors. However, the study from Hsieh et al. (2015) has indicated that errors occurred more frequently with lower No-go probability than higher No-go probability. A more demanding tendency of inhibition, i.e., lower No-go probability, might be more sensitive to the age-related inhibitory deficit. Besides, we found that the senior’s RT was comparable to the younger’s RT, while most of the earlier studies have shown a delayed RT in the older adults (Vallesi et al., 2009; Vallesi, 2011; Hong et al., 2014; Hsieh et al., 2015; Mudar et al., 2015). Our results were supported by the speed-accuracy trade-off theory (Hoffmann and Falkenstein, 2011; Endrass et al., 2012). It was suggested that the seniors spent longer RT in order to reduce response errors. In other words, seniors who exhibit RT similar to or faster than younger adults’ would show higher error rates.

The Go/No-go task through MEG recordings was only studied in adolescents and young adults (Vidal et al., 2012; Vara et al., 2014). Age-related activation changes in response inhibition by using MEG examination remained less elucidated. According to the spatiotemporal maps of brain activation, a more frontal distribution was observed in the older adults than in the younger. Further analysis showed that the seniors had a delayed activation in PFC, pre-SMA and MTG, as well as a declined cortical amplitude in MTG. Our results were consistent with previous studies that prolonged latency was observed due to inhibition deficits in the older adults (Tachibana et al., 1996; Bokura et al., 2002; Falkenstein et al., 2002; Vallesi, 2011; Hsieh et al., 2015, 2016; Mudar et al., 2015). It was interesting to note that the strength of MTG activation was significantly reduced in the older adults. Several lines of evidence have shown the role of left MTG in the verbal retrieval of semantic information (Martin and Chao, 2001; Fiebach et al., 2002). In our current study, the stimuli were varied in font size to prevent bottom-up attentional bias from top-down inhibitory control. Thus, the participants had to give a response judged by naming numbers instead of visual features. Regarding the number processing, Dehaene’s model has shown that the numerals, both Arabic digits and verbal number words, would be converted into the semantic magnitude representation (Dehaene and Akhavein, 1995). The internal representation is recognized at the left parietal areas, and then transmitted to the left-hemispheric perisylvian language areas (e.g., middle and superior temporal gyrus, supramarginal gyrus, etc.) for naming (Dehaene and Cohen, 1997; Klein et al., 2014). More importantly, it has been proposed that either mathematical problem solving or number naming is mediated by sematic representation (Fias et al., 2001; Ashkenazi et al., 2012; Zhou et al., 2018). A recent study with neural network analysis has suggested that MTG, particularly the posterior division, and IPL were connected with the ventrolateral PFC, an area considered to be involved in the higher-level cognitive control for the semantic information (Davey et al., 2016). Thus, we reasoned that the reduced activation of MTG in the older adults was correlated with the altered semantic-related processing. Semantic cognition has been proposed to consist of two components. One is the semantic representation that provides conceptual meanings to verbal or nonverbal experiences; another is semantic control processes which detect the target message to guide context-proper behaviors (Jefferies and Lambon Ralph, 2006; Jefferies, 2013; Davey et al., 2016). The MTG was considered to contribute to the semantic control by identifying the information to guide behaviors (Noonan et al., 2013). Hence, reduced MTG activities in the older adults may suggest an age-related decline in the integrity of the semantic control processing (Jefferies, 2013; Butorina et al., 2017). Further research is needed to examine whether age-related deficits in the left temporal regions are related to the deficient control processing of semantic retrieval.

Our results showed that low-performers responded to Go stimuli faster than high-performers did. The finding was consistent with previous results, suggesting that speeding in responses was driven by the prepotent tendency, resulting in inefficiently withholding responses (Cheyne et al., 2009; Hoffmann and Falkenstein, 2011). The results were also in line with the speed-accuracy trade-off theory where shorter RT to Go trials in low-performers would lead to higher error rates (Hoffmann and Falkenstein, 2011; Endrass et al., 2012). However, the differences in response inhibition between the high-performers and low-performers could not be attributed to different attentional levels since the miss rate was equivalent between the two subgroups. Thus, our data, together with the previous findings, indicated that low-performers had more difficulty in inhibiting their responses to No-go stimuli.

Moreover, we found that the high-performers utilized greater neural sources in the PFC, especially at the left side, than the low-performers did. The phenomenon of frontal over-recruitment in our current study may be in accord with the compensation hypothesis between high- and low-performing older adults in terms of memory tasks (Cabeza et al., 2002; Lubitz et al., 2017). There were similar findings in the previous studies. For example, Hong et al. (2014) have examined age-associated neural responses to inhibition-related ERP components, showing that compared to younger adults, older adults had more brain activation during successful inhibition. Another earlier report has also indicated that older adults with over-recruitment of neural substrates tended to attain better performance (Vallesi et al., 2011). Moreover, Nielson et al. (2002) have found that the seniors recruited more ipsilateral and contralateral PFC, as well as parietal regions to assist with inhibitory performance. In contrast, our finding is inconsistent with another view that over-recruitment was considered to reflect inefficient processing. The interpretation was proposed that the aging brain exhibited reduced specific cortical activities (i.e., dedifferentiation) and increased more cortical activation widespread to achieve the equivalent performance of the younger brain (Reuter-Lorenz and Cappell, 2008; Reuter-Lorenz and Park, 2010). In our present findings, the phenomenon of over-recruitment was found in the high performers instead of the low performers among the older adults. Our result supported this compensation view.

Furthermore, some studies have reported that response inhibition consistently activated the right-lateralized prefrontal regions (Aron et al., 2004, 2014; Chikazoe, 2010), while some noted that the left side of PFC also contributes to the processes of inhibitory control (Aron et al., 2004, 2014; Swick et al., 2008). A tempting explanation is that the high-performers, compared to the low-performers, may deploy different strategies to perform Go/No-go tasks. It has been shown that increased activation of left PFC may contribute to greater demands in decision operations (Green et al., 2016; Gagnepain et al., 2017). The left PFC activation were also proposed to be involved in the control processes of decision making and evaluation (Xue et al., 2012; Turi et al., 2015). Moreover, the left PFC activation may be accompanied by increased attentional processes to reflect a more controlled resolution to response execution (Nee et al., 2007). Thus, the stronger left PFC activation in the high-performers may assist in the resolution of the interference and the selection of semantic targets during the inhibitory processes. Taken together, our data suggested that the high-performing elderly had additional activation of resources (i.e., left PFC) to cope with task demands, leading to a better performance of response inhibition.

Several limitations should be addressed. First, due to the limited sample size in this study, it might increase the risk of bias in the interpretation of neural activation between the older subgroups. Second, taking the education level into account could better elucidate the age-related alterations regarding their cognitive performance and brain responses. Although the education level was lower in the older group than in the younger group, we utilized the standardized cognitive measurement to confirm that the older subjects had normal cognitive function. Finally, it might be intriguing to examine the potential factors leading to the differences of brain activations between high- and low-performers among the older participants. However, the small sample size and the insufficient demographic data would impede us to perform this investigation.

In conclusion, age-related deficiencies of response inhibition were observed from both behavioral performance and neurophysiological measurement. Compared to the younger adults, the older adults had more FA errors and delayed recruitment of cortical activities to inhibit unnecessary responses. Besides, more left frontal activation was found in high-performers than in low-performers among the older participants. That is, the frontal recruitment may play a compensatory role in successful inhibition.

## Author Contributions

M-YL and C-HC conceived and designed the work and wrote the article. M-YL acquired the data and analyzed the data. M-YL, Y-JT and C-HC participated in the discussion and provided the comments. All of the authors have read and approved the manuscript.

## Conflict of Interest Statement

The authors declare that the research was conducted in the absence of any commercial or financial relationships that could be construed as a potential conflict of interest.
